# Hospital Admissions and Antimicrobial Resistance Patterns in Diabetic Patients With Urinary Tract Infections: A Cross-Sectional Study From a Tertiary Care Center in Pakistan

**DOI:** 10.7759/cureus.89432

**Published:** 2025-08-05

**Authors:** Ali Raza, Numan Pervaiz, Irfan Ali, Mariam bint Shafqat, Muhammad Zubair, Misbah Shirin Memon, Abdullah Shaikh, Hafiza Sobia Ramzan

**Affiliations:** 1 Department of Medicine, Gulab Devi Teaching Hospital, Lahore, PAK; 2 Department of Medicine, Farooq Hospital, Lahore, PAK; 3 Department of Cardiology, Gulab Devi Teaching Hospital, Lahore, PAK; 4 Department of General Medicine, Pakistan Institute of Medical Sciences, Islamabad, PAK; 5 Department of Urology, Indus Medical College Hospital, Tando Mohammad Khan, PAK; 6 Department of Biochemistry, Institute of Molecular Biology and Biotechnology, University of Lahore, Lahore, PAK

**Keywords:** diabetes milletus, glycemic control, infection, risk factors, uti

## Abstract

Background

Diabetes mellitus is a global public health challenge, significantly increasing susceptibility to infections, particularly urinary tract infections (UTIs). Diabetic patients face a higher risk of recurrent and complicated UTIs due to impaired immune function, poor glycemic control, and associated comorbidities.

Objective

This study aimed to determine the frequency and demographic trends of hospital admissions in diabetic patients suffering from urinary tract infections, identify associated clinical risk factors, evaluate the microbiological profile of uropathogens, and assess prevailing patterns of antimicrobial resistance.

Methodology

This cross-sectional study was conducted at Gulab Devi Hospital, Lahore, from 1st January 2025 to 30th June 2025. A total of 325 diabetic patients admitted with suspected UTIs were included using non-probability consecutive sampling. Demographic, clinical, and laboratory data were collected. Midstream urine samples were cultured to identify pathogens, and antimicrobial susceptibility was tested using the disc diffusion method according to Clinical and Laboratory Standards Institute (CLSI) guidelines.

Results

Among 325 participants, 205 patients (63.1%) had culture-confirmed UTIs. The mean age of UTI-positive patients was 59.2 ± 9.8 years, significantly higher than non-UTI patients (55.8 ± 11.3 years; p = 0.01). Age over 60 years was present in 129 (62.9%) UTI cases. Poor glycemic control (HbA1c >8%) was found in 162 (79.0%) UTI patients, and 152 (74.1%) had diabetes duration exceeding 10 years. Hypertension was reported in 148 (72.2%) and diabetic nephropathy in 67 (32.7%) UTI patients. *Escherichia coli* was the predominant pathogen in 117 (57.1%) cases, followed by *Klebsiella pneumoniae* in 37 (18.0%) cases. High resistance rates were seen for amoxicillin in 154 (75.1%) and cotrimoxazole in 139 (67.8%) isolates, while nitrofurantoin and ceftriaxone demonstrated high sensitivity in 174 (84.9%) and 166 (81.0%) cases, respectively.

Conclusion

It is concluded that urinary tract infections are a common and serious cause of hospital admissions in diabetic patients, particularly those with advanced age, poor glycemic control, and long-standing diabetes. *Escherichia coli *remains the predominant pathogen with evolving antimicrobial resistance trends.

## Introduction

Diabetes mellitus (DM) is one of the most prevalent non-communicable diseases globally, with an increasing trend in both developed and developing countries [1]. Roughly 537 million people worldwide aged 20-79 years were living with diabetes in 2021, and estimates suggest that this number could grow to 643 million by the year 2030 (International Diabetes Federation, IDF Diabetes Atlas 2021) [2].

In South Asia, urbanization, a marked increase in physical inactivity, dietary habits, and genetics make the region a hotspot for diabetes. With approximately 33 million affected adults, Pakistan ranks 10th in the world in diabetes prevalence according to the National Diabetes Survey of Pakistan 2016-17 [3].

A noteworthy and potentially serious complication of diabetes is urinary tract infections (UTIs). Diabetics face a two to four times greater risk of developing UTIs compared to non-diabetics [4]. This condition occurs due to numerous reasons that include immune system complications due to hyperglycemia, weakened neutrophils, bladder emptying issues due to autonomic neuropathy, and high sugar levels in urine that lead to bacterial proliferation [5]. Furthermore, diabetic nephropathy and other urinary tract structural changes create easy pathways for recurrent UTIs. UTIs are well known as common causes of morbidity and hospitalizations among patients with diabetes, as evidenced from clinical observations. Research indicates that as many as 25-30% of hospital admissions for individuals with diabetes may be due to infections, a significant portion of which are urinary tract infections (UTIs).

Within the Pakistani healthcare framework, diabetic patients with infectious comorbidities are routinely managed in diabetic clinics of tertiary care hospitals such as Gulab Devi Hospital [6]. Even so, there appears to be a distinct absence of systematically collected local data on the prevalence and trends of diabetes-related urinary tract infection hospitalizations [7].

The effects of urinary tract infections among patients with diabetes go beyond simple infection management. In tandem with poor glycemic control, pre-existing infections can lead to complicated pyelonephritis, renal abscess, emphysematous cystitis, or even urosepsis, which is known for its high mortality rate [8]. There is also the problem of recurrent hospital admissions, which impacts the socioeconomic burden of healthcare, hospital resource allocation, and the overall quality of life for the patients [9]. In the context of Pakistan, where the healthcare system is already strained with chronic and communicable diseases, an understanding of these patterns is important to refine patient pathways and develop targeted preventive interventions. Emerging patterns of evolving antimicrobial resistance among uropathogens in diabetic patients have also been noted in newer studies [10]. Treatment regimens are further complicated due to increasing resistant bacterial strains, such as *Escherichia coli*, *Klebsiella pneumoniae*, and *Enterococcus *species, to commonly prescribed antibiotics [11]. 

Objective

This study was conducted to address this gap by evaluating the frequency and demographic distribution of hospital admissions due to UTIs in diabetic patients, identifying key clinical risk factors, and analyzing both the spectrum of causative bacterial pathogens and their antimicrobial susceptibility profiles.

## Materials and methods

This was a cross-sectional study conducted at Gulab Devi Hospital, Lahore, over a period of six months from 1st January 2025 to 30th June 2025, including 325 diabetic patients with suspected urinary tract infections, selected through non-probability consecutive sampling.

Inclusion criteria

Patients eligible for the study were diabetic individuals aged between 18 and 80 years. Only those with a confirmed diagnosis of urinary tract infection (UTI), based on clinical symptoms such as dysuria, frequency, urgency, or suprapubic discomfort, along with a positive urine culture, were included. Both type 1 and type 2 diabetes mellitus patients were enrolled by the diagnostic criteria outlined by the American Diabetes Association (ADA).

Exclusion criteria

Patients were excluded from the study if they had chronic renal failure or were undergoing dialysis, as these conditions could confound the analysis of infection severity and outcomes. Individuals with indwelling urinary catheters or who had undergone recent urological interventions were also excluded due to the elevated risk of non-community-acquired infections. Furthermore, patients with known immunocompromised conditions, such as HIV/AIDS or those on long-term corticosteroid therapy, were not included.

Data collection

After obtaining ethical approval from the Gulab Devi Hospital Ethical Review Board and informed consent from all participants, data were collected from 325 diabetic patients who were admitted with symptoms suggestive of UTI. Each participant underwent a thorough clinical examination. Demographic details such as age, gender, type and duration of diabetes, and comorbid conditions were recorded. Relevant history regarding frequency of UTIs, previous hospitalizations, and history of antimicrobial use was noted. A midstream urine sample was collected in sterile containers. The samples were processed in the hospital’s microbiology laboratory, where urine cultures were performed to identify bacterial pathogens. The isolated organisms were subjected to antimicrobial susceptibility testing using the disc diffusion method according to Clinical and Laboratory Standards Institute (CLSI) guidelines. Additional laboratory investigations, including fasting blood glucose, HbA1c levels, renal function tests, and complete blood counts, were documented where available. The length of hospital stays and treatment outcomes (such as discharge, readmission, or in-hospital mortality) were also recorded for each patient.

Statistical analysis

The data were analyzed using SPSS version 26 (IBM Corp., Armonk, USA). Descriptive statistics were applied to summarize continuous variables such as age, duration of diabetes, and hospital stay, which were reported as mean ± standard deviation. Categorical variables such as gender, type of diabetes, urine culture positivity, and bacterial isolates were expressed as frequencies and percentages. Chi-square tests were performed to examine associations between categorical variables, including age groups, gender, type of diabetes, and frequency of UTI-related hospital admissions. A p-value of <0.05 is statistically significant.

## Results

Patients with UTI had a slightly higher mean age (59.2 ± 9.8 years) compared to those without UTI (55.8 ± 11.3 years), suggesting age as a contributing factor. Mean BMI values were similar between groups (27.4 ± 4.3 kg/m² in UTI vs. 27.0 ± 4.0 kg/m² in non-UTI). The prevalence of type 2 diabetes mellitus was higher in the UTI group (90% vs. 84%), with a notably longer mean diabetes duration in UTI patients, reflected by 109 (53%) having diabetes for over 10 years compared to 40 (33%) in non-UTI patients. Poor glycemic control (HbA1c > 8%) was also more frequent in the UTI group (63% vs. 49%) (Table 1).

**Table 1:** Demographic and Clinical Characteristics of Participants

Characteristic	Total (n=325)	UTI Group (n=205)	No UTI (n=120)
Age (years)	58.4 ± 10.6	59.2 ± 9.8	55.8 ± 11.3
Gender (Male)	62% (201/325)	60% (123/205)	65% (78/120)
BMI (kg/m²)	27.2 ± 4.1	27.4 ± 4.3	27.0 ± 4.0
Type 2 Diabetes Mellitus	88% (286/325)	90% (185/205)	84% (101/120)
Duration of Diabetes (>10 yrs)	46% (149/325)	53% (109/205)	33% (40/120)
HbA1c > 8%	58% (188/325)	63% (129/205)	49% (59/120)
Bacteria			
Escherichia coli	57.1% (117/205)	57.1% (117/205)	0% (0/120)
Klebsiella pneumoniae	18.0% (37/205)	18.0% (37/205)	0% (0/120)
Proteus mirabilis	9.3% (19/205)	9.3% (19/205)	0% (0/120)
Enterococcus faecalis	8.2% (17/205)	8.2% (17/205)	0% (0/120)
Pseudomonas aeruginosa	7.3% (15/205)	7.3% (15/205)	0% (0/120)

Among 117 *Escherichia coli *isolates, susceptibility was highest to nitrofurantoin 100 (85%), followed by ceftriaxone 95 (81%), ciprofloxacin 82 (70%), gentamicin 87 (74%), amoxicillin 88 (75%), and cotrimoxazole 80 (68%). For 37 *Klebsiella pneumoniae *isolates, nitrofurantoin showed 32 (87%) susceptibility, followed by ceftriaxone 31 (83%), gentamicin 25 (68%), ciprofloxacin 22 (60%), amoxicillin 22 (60%), and cotrimoxazole 20 (54%). *Proteus mirabilis *(n = 19) showed nitrofurantoin susceptibility in 17 (91%), ceftriaxone in 16 (85%), gentamicin in 14 (72%), amoxicillin in 13 (66%), ciprofloxacin in 12 (65%), and cotrimoxazole in 11 (58%) (Table 2).

**Table 2:** Antimicrobial Susceptibility Pattern of Isolates

Antibiotic	*Escherichia coli *(%)	*Klebsiella pneumoniae *(%)	*Proteus mirabilis *(%)
Amoxicillin	75% (88/117)	60% (22/37)	66% (13/19)
Cotrimoxazole	68% (80/117)	54% (20/37)	58% (11/19)
Nitrofurantoin	85% (100/117)	87% (32/37)	91% (17/19)
Ceftriaxone	81% (95/117)	83% (31/37)	85% (16/19)
Gentamicin	74% (87/117)	68% (25/37)	72% (14/19)
Ciprofloxacin	70% (82/117)	60% (22/37)	65% (12/19)

Age over 60 years was noted in 82 (40%) UTI patients and 36 (30%) non-UTI patients (p = 0.01). HbA1c > 8% was found in 129 (63%) UTI patients compared to 59 (49%) non-UTI patients (p = 0.005). Diabetes duration over 10 years was present in 109 (53%) UTI patients and 40 (33%) non-UTI patients (p < 0.001). Hypertension was observed in 146 (71%) UTI patients versus 65 (54%) non-UTI patients (p = 0.002). Diabetic nephropathy was recorded in 45 (22%) UTI patients and 14 (12%) non-UTI patients (p = 0.03) (Table 3).

**Table 3:** Risk Factors for UTI in Diabetic Patients A p-value < 0.05 was considered statistically significant. DM: diabetes mellitus; UTI: urinary tract infection

Risk Factor	UTI Group (n = 205)	No UTI Group (n = 120)	χ² (df)	p-value
Age > 60 years	82 (40.0%)	36 (30.0%)	6.45 (1)	0.01
HbA1c > 8%	129 (62.9%)	59 (49.2%)	7.91 (1)	0.005
Duration of DM > 10 years	109 (53.2%)	40 (33.3%)	13.09 (1)	<0.001
Hypertension	146 (71.2%)	65 (54.2%)	9.81 (1)	0.002
Diabetic Nephropathy	45 (22.0%)	14 (11.7%)	4.83 (1)	0.03

Hospital stay over 7 days was recorded in 59 (29%) UTI patients versus 17 (14%) non-UTI patients (p < 0.001). Recurrent admissions occurred in 37 (18%) UTI patients compared to 11 (9%) in the non-UTI group (p = 0.02). In-hospital mortality was higher among UTI patients at 13 (6%) compared to three (2%) non-UTI patients (p = 0.04). Acute kidney injury was documented in 21 (10%) UTI patients and five (4%) non-UTI patients (p = 0.03) (Table 4).

**Table 4:** Comparison of Hospital Stay and Complications Chi-square (χ²) test was used to compare categorical outcomes between UTI and non-UTI groups. Degrees of freedom (df) = 1 for each comparison. p-values < 0.05 indicate statistically significant associations. UTI: urinary tract infection

Outcome	UTI Group (n = 205)	No UTI Group (n = 120)	χ² (df)	p-value
Hospital Stay > 7 days	59 (28.8%)	17 (14.2%)	10.39 (1)	<0.001
Recurrent Admission	37 (18.0%)	11 (9.2%)	5.36 (1)	0.02
In-hospital Mortality	13 (6.3%)	3 (2.5%)	4.10 (1)	0.04
Acute Kidney Injury (AKI)	21 (10.2%)	5 (4.2%)	4.59 (1)	0.03

Age > 60 years increased the odds of UTI with an OR of 2.4 (95% CI: 1.5-3.9, p = 0.01). HbA1c > 8% was associated with an OR of 2.8 (95% CI: 1.7-4.4, p = 0.002). Diabetes duration over 10 years had the strongest association with an OR of 3.1 (95% CI: 1.9-5.0, p < 0.001). Hypertension had an OR of 1.9 (95% CI: 1.2-3.0, p = 0.03), and diabetic nephropathy showed an OR of 1.7 (95% CI: 1.0-2.9, p = 0.04) (Table 5).

**Table 5:** Multivariate Logistic Regression for Predictors of UTI in Diabetic Patients All predictors are statistically significant at p < 0.05. Odds ratios (ORs) with 95% confidence intervals (CI) are presented. DM: diabetes mellitus

Predictor	Odds Ratio (95% CI)	Wald χ² (df)	p-value
Age > 60 years	2.4 (1.5–3.9)	6.47 (1)	0.01
HbA1c > 8%	2.8 (1.7–4.4)	9.62 (1)	0.002
Duration of DM > 10 years	3.1 (1.9–5.0)	13.77 (1)	<0.001
Hypertension	1.9 (1.2–3.0)	4.67 (1)	0.03
Diabetic Nephropathy	1.7 (1.0–2.9)	4.04 (1)	0.04

## Discussion

This study was conducted to evaluate the frequency and trends of hospital admissions in diabetic patients suffering from urinary tract infections (UTIs) at Gulab Devi Hospital, Lahore. These findings show clearly that diabetic patients with UTIs have a unique clinical presentation; the findings show older age, prolonged diabetes duration, ineffective glycemic regulation, and other clinically relevant comorbidities in the form of hypertension and diabetic nephropathy. The age of the sample involved in this study had a mean of 58.4 years, with a greater variance in the mean age of UTI-positive patients than that of non-UTI diabetic ones. This finding is in line with the past literature that has always stated that descriptively, aged diabetics are more prone to UTIs owing to the occurrence of age-related immune impairment and bladder dysfunction [12]. The present study supports similar findings in older age above 60 years, having an independent risk factor (odds ratio) of 2.4. Another outstanding risk factor was the duration of diabetes, where over 53% of UTI-positive patients had diabetes for over 10 years. This observation roughly correlates with previous studies' findings, in which long-term diabetes is associated with a higher chance of infection as a result of progressive metabolism disorder, persistent hyperglycemia, and autonomic neuropathy of the urinary system [13,14].

Noticeable poor glycemic control was present in 63% of UTIs, and an HbA1c level of over 8% was present. This agrees with the earlier studies, which indicated that high levels of HbA1c hinder the functioning of neutrophils, promoting the proliferation of bacteria in the urinary tract, resulting in asymptomatic bacteriuria, as well as symptomatic UTIs. The logistic regression model used in the present study also proved that HbA1c greater than 8% was an independent predictor of UTI, again showing similar trends to earlier studies [15]. *Escherichia coli* was found as the most prevalent uropathogen (57.1%), followed by *Klebsiella pneumoniae* (18%) and *Proteus mirabilis *(9.3%). The current distribution is very much similar to past research outcomes, whereby *E. coli *has been reported as the most dominant pathogen in diabetic UTI all over the world. The higher *Klebsiella* and *Proteus *species rates compared to non-diabetic groups evidenced here also compare with the literature as presented by past articles, probably because diabetic people have more complicated profiles of the infections [16,17]. With respect to antibiotic resistance patterns, the study recorded high resistance percentage against amoxicillin and cotrimoxazole, whereas nitrofurantoin and ceftriaxone showed excellent efficacy against the majority of isolates. This reflects emerging trends in antimicrobial susceptibilities that were presented in the past studies carried out in similar tertiary care institutions due to rising empirical prescribing of broad-spectrum antibiotics. Resistance to nitrofurantoin is vital to note, especially since previous studies indicated that it could be used as a first-line drug against uncomplicated UTIs among diabetic patients [18].

The result of the clinical outcomes of this study showed that diabetic patients with UTIs took more time to be discharged, had a greater rate of readmission, a higher mortality rate, and comorbidities like acute kidney injury. In particular, 29% of UTI patients spent more than 7 days in the hospital, and mortality in the hospital was 6% as compared to 2% in non-UTI patients. Such numbers are comparable to what has been reported in other studies, which speaks of the high clinical and economic impact of UTI-related hospitalization in diabetic groups [19]. Age, poor glycemic control, duration of diabetes, hypertension, and diabetic nephropathy were found to be independent predictors of UTI by the multivariate logistic regression. Such a multifactorial risk profile fully complies with the results of the previous studies that highlighted that UTI risk in patients with diabetes does not depend on one and only factor but on an interaction of demographic, metabolic, and comorbid circumstances [20].

Limitations

This study has several limitations that should be considered when interpreting the findings. To begin with, the non-probability selection consecutive sampling has a possibility of involving bias since the sampled group cannot universally represent the larger groups of diabetics. Second, the research was carried out in one tertiary care facility, thus limiting the extent to which the findings can be generalizable to other care facilities or to other regions with varied demographic and clinical dynamics. Also, formal sample size estimation and power estimation were not done before the data were collected; this possibly influences the statistical accuracy of the estimates made in some cases. The research study was conducted over a period of six months, so it might not capture any seasonal changes in the rate of urinary tract infection, microbial resistance, and admission rate. Finally, we could not follow up changes in the resistance patterns of recurrent or nosocomial infections since the study only involved the first isolate to be microbiologically analyzed. Despite these limitations, the study provides valuable baseline data on the risk factors, microbial profiles, and antimicrobial resistance patterns associated with urinary tract infections in hospitalized diabetic patients.

## Conclusions

In conclusion, urinary tract infections represent a significant cause of hospital admissions among diabetic patients, particularly those with older age, longer duration of diabetes, poor glycemic control, hypertension, and diabetic nephropathy. *Escherichia coli *was identified as the most common causative organism, with notable resistance patterns against commonly used antibiotics such as amoxicillin and cotrimoxazole. Patients with UTIs experienced longer hospital stays, higher readmission rates, and increased risk of complications, including acute kidney injury and mortality.
